# 24-Month Clinical, Immuno-Virological Outcomes, and HIV Status Disclosure in Adolescents Living With Perinatally-Acquired HIV in the IeDEA-COHADO Cohort in Togo and Côte d'Ivoire, 2015–2017

**DOI:** 10.3389/fped.2021.582883

**Published:** 2021-06-30

**Authors:** Marc Harris Dassi Tchoupa Revegue, Unoo Elom Takassi, François Tanoh Eboua, Sophie Desmonde, Ursula Belinda Amoussou-Bouah, Tchaa Abalo Bakai, Julie Jesson, Désiré Lucien Dahourou, Karen Malateste, Hortense Aka-Dago-Akribi, Jean-Philippe Raynaud, Elise Arrivé, Valériane Leroy

**Affiliations:** ^1^Center for Epidemiology and Research in POPulation Health (CERPOP), Inserm, Université de Toulouse, Université Paul Sabatier, Toulouse, France; ^2^Department of Pediatrics, Centre Hospitalier Universitaire Sylvanus Olympio, Lomé, Togo; ^3^Department of Pediatrics, Centre Hospitalier Universitaire de Yopougon, Abidjan, Côte d'Ivoire; ^4^Département Biomédical et de Santé Publique, Institut de Recherche en Sciences de la Santé (IRSS/CNRST), Ouagadougou, Burkina Faso; ^5^Centre Muraz, Bobo-Dioulasso, Burkina Faso; ^6^Inserm U1219-Epidemiologie-Biostatistique, Université de Bordeaux, Bordeaux, France; ^7^Département de Psychologie Clinique, Université Houphouet-Boigny, Abidjan, Côte d'Ivoire; ^8^Service Universitaire de Psychiatrie de l'Enfant et de l'Adolescent, CHU de Toulouse, Toulouse, France

**Keywords:** adolescents, HIV, disclosure, retention, West-Africa

## Abstract

**Background:** Adolescents living with perinatally-acquired HIV (APHIV) face challenges including HIV serostatus disclosure. We assessed their 24-month outcomes in relation to the disclosure of their own HIV serostatus.

**Methods:** Nested within the International epidemiologic Database to Evaluate AIDS pediatric West African prospective cohort (IeDEA pWADA), the COHADO cohort included antiretroviral (ART)-treated APHIV aged 10–19 years, enrolled in HIV care before the age of 10 years, in Abidjan (Côte d'Ivoire) and Lomé (Togo) in 2015. We measured the HIV serostatus disclosure at baseline and after 24 months and analyzed its association with a favorable combined 24-month outcome using logistic regression. The 24-month combined clinical immuno-virological outcome was defined as unfavorable when either death, loss to follow-up, progression to WHO-AIDS stage, a decrease of CD4 count >10% compared to baseline, or a detectable viral load (VL > 50 copies/mL) occurred at 24 months.

**Results:** Overall, 209 APHIV were included (51.6% = Abidjan, 54.5% = females). At inclusion, the median CD4 cell count was 521/mm^**3**^ [IQR (281–757)]; 29.6% had a VL measurement, of whom, 3.2% were virologically suppressed. APHIV were younger in Lomé {median age: 12 years [interquartile range (IQR): 11–15]} compared to Abidjan [14 years (IQR: 12–15, *p* = 0.01)]. Full HIV-disclosure increased from 41.6% at inclusion to 74.1% after 24 months. After 24 months of follow-up, six (2.9%) died, eight (3.8%) were lost to follow-up, and four (1.9%) were transferred out. Overall, 73.7% did not progress to the WHO-AIDS stage, and 62.7% had a CD4 count above (±10%) of the baseline value (48.6% in Abidjan vs. 69.0% in Lomé, *p* < 0.001). Among the 83.7% with VL measurement, 48.8% were virologically suppressed (Abidjan: 45.4%, Lomé: 52.5%, *p* <0.01). The 24-month combined outcome was favorable for 45% (29.6% in Abidjan and 61.4% in Lomé, *p* < 0.01). Adjusted for baseline variables, the 24-month outcome was worse in Lomé in those who had been disclosed for >2 years compared to those who had not been disclosed to [aOR = 0.21, 95% CI (0.05–0.84), *p* = 0.03].

**Conclusions:** The frequency of HIV-disclosure improved over time and differed across countries but remained low among West African APHIV. Overall, the 24-month outcomes were poor. Disclosure before the study was a marker of a poor 24-month outcome in Lomé. Context-specific responses are urgently needed to improve adolescent care and reach the UNAIDS 90% target of virological success.

## Introduction

Access to antiretroviral therapy (ART) in sub-Saharan Africa has significantly expanded since 2004 (1). As a result, children living with perinatally-acquired HIV are now growing up into adolescence. The critical period of adolescence is characterized by biological and psychosocial changes in a context where HIV disease is turning into a chronic condition (2, 3). Globally, 1.8 million adolescents aged 10–19 years were living with HIV in 2017 (4). Sub-Saharan Africa is the most impacted region, accounting for 84% of the adolescents living with HIV (ALHIV) (5–7); adolescent girls accounted for three-quarters of all new HIV infections among adolescents in 2018 (8). HIV/AIDS was the leading cause of death amongst adolescents in this region in 2016 (9). Among the 23 African priority countries, the estimated AIDS-related deaths were 24,000 (17,000–33,000) in adolescents aged 10–14 years and 25,000 (18,000–34,000) in those aged 15–19 years in 2017 (10). Despite the progress achieved in pediatric HIV care, attention must be paid to this expanding population of adolescents living with either perinatally or non-perinatally acquired HIV, since it is estimated that new HIV infections in adolescents will increase 13% annually by 2030 in Africa (11, 12). This is particularly true in West and Central Africa, which has recorded a 35% increase in the annual number of AIDS-related deaths among adolescents aged 15–19 years from 2010 to 2016 (13).

Since 2015, universal ART is recommended by the WHO (14). Compared to adults or younger children, adolescents living with perinatally acquired HIV (APHIV) experience a higher morbidity and mortality and lower rates of virological suppression on ART (3, 15–17). This is most likely related to the delayed access to HIV diagnosis and ART in childhood, lack of timely HIV-disclosure while growing-up, with poor medication adherence in a context of prolonged ART, poor retention in care, explained by individual, social, and structural barriers (7, 18–22). Transition to adult care also remains a vulnerable step in ALHIV care (23, 24). All these factors contribute to delaying the UNAIDS' 90-90-90 targets in the ALHIV cascade of care (25).

However, a timely HIV-disclosure in APHIV is a crucial step to motivate ART adherence and to achieve these 90-90-90 UNAIDS targets (22, 25). Full disclosure of an HIV diagnosis includes naming HIV/AIDS and providing information about care and the modes of HIV transmission (22). Unfortunately, a large proportion of APHIV diagnosed during their infancy remains unaware of their HIV-positive status while growing up. In many West African pediatric clinical sites, even if children or adolescents are on ART, healthcare providers and caregivers delay HIV disclosure because of cultural factors and lack of national guidance (22). Also, caregivers are not ready and fear that the process will lead to disclosing a family secret with subsequent stigma (26–29). According to a previous review, 1.7–41% of children and ALHIV in low- and middle-income countries have received full disclosure of their HIV-positive status (30). In West Africa, less than a third of APHIV knew their HIV status in 2011, and the HIV disclosure process often occurred late after the WHO-recommended age of 12 years (31, 32). Data suggests that an earlier full HIV disclosure could improve ALHIV outcomes through improved ART adherence, retention in care, and slower disease progression (29, 33, 34).

The prospective monitoring of the HIV disclosure process in APHIV is crucial to understand their clinical, immunological, and virological long-term outcomes. To better document the outcomes of APHIV in West Africa, the COHADO (“COHort of ADOlescents living with HIV”) sub-cohort, as part of the West African International epidemiologic Database to Evaluate AIDS (IeDEA) pediatric cohort Collaboration, was launched in 2015 in two sites of the West African IeDEA pediatric cohort in Lomé (Togo) and Abidjan (Côte d'Ivoire). This study is aimed to better document HIV disclosure frequency and process over 24 months of follow-up; and to assess the association of HIV disclosure with the 24-month health outcomes among APHIV since their inclusion in the COHADO cohort.

## Methods

### Study Design

The IeDEA pediatric West African Database to evaluate AIDS (pWADA) is an international multicentric prospective cohort as part of the IeDEA global pediatric collaboration (https://www.iedea.org/), supported by the US National Institutes of Health since 2006, to describe HIV epidemiology trends and evaluate HIV outcomes using large patient-level observational databases. It includes children and adolescents living with HIV from 11 pediatric clinical centers in seven West African countries (Benin, Burkina Faso, Côte d'Ivoire, Ghana, Mali, Senegal, and Togo) and is aimed at addressing HIV/AIDS research questions regarding HIV care and outcomes in children and adolescents living with HIV in West Africa (35). Nested in pWADA, the COHADO prospective cohort was a pilot study aimed to explore the feasibility of collecting prospective data to specifically focus on APHIV issues such as the HIV-disclosure process and behavioral issues in two pilot sites, Lomé (Togo) and Abidjan (Côte d'Ivoire), between 2015 and 2017.

### Settings and Study Population

The COHADO cohort included APHIV from the pediatric pWADA active file of the Teaching Hospital of Yopougon (Abidjan, Côte d'Ivoire) and the Sylvanus Olympio Teaching Hospital (Lomé, Togo). In accordance with the national guidelines, APHIV followed-up in these sites are typically seen in medical consultation on a quarterly basis. CD4 count were measured 6-monthly, and, since 2016, viral load is monitored on a yearly basis.

Between January and November 2015, ART-treated ALHIV aged 10–19 years, included in HIV care before the age of 10 years (as a proxy of perinatal infection in the absence of a documented mode of transmission) and followed up in the two participating sites, were invited to enroll in the study.

### Ethics Approval and Consent to Participate

This study has received authorizations from the health ministries and national ethics committee of Togo and Côte d'Ivoire. All the participants and their caregivers gave their written informed consent to participate in the COHADO cohort. A specific consent form was adapted to the adolescents' awareness of their HIV status to avoid any unintentional disclosure.

### Data Collection

Study-specific data including living and schooling conditions, parents' vital status, access to running water, access to electricity, disclosure (at baseline and during follow-up), and disclosure process were recorded using standardized questionnaires which are assessed yearly. Other sociodemographic, clinical, and therapeutic data were extracted from medical records and the IeDEA pediatric central database. Since the causes of death were not documented, we hypothesized that all deaths were HIV-related.

The word HIV was not mentioned in the adolescent questionnaires to avoid any accidental HIV disclosure to the adolescent during the interview. Data on the HIV disclosure process (disclosure status, date of full disclosure if delivered over the cohort period, person in charge of disclosure) were collected from the parents/legal guardian, rather than the adolescents themselves. HIV disclosure was defined as full disclosure when the adolescent was specifically told that he or she has HIV/AIDS and he/she knew the care and modes of transmission (22).

Treatment adherence was evaluated by the counselor using the ratio between the number of pills prescribed and the number of pills missed in the last days preceding the visit and declared by the adolescent.

### Data Analysis

Baseline was defined as the date of inclusion in the COHADO cohort.

First, among the eligible APHIV, we compared the baseline characteristics of those who were included vs. those who were not included in the study, using median values with interquartile ranges (IQR) for continuous variables and proportions for categorized variables, overall and by site. Full HIV disclosure was assessed at baseline, and then over the 24-month follow-up period. APHIV were classified at 24 months as not HIV-disclosed to, disclosed to during the past 24 months (after inclusion in COHADO), or HIV-disclosed to for more than 2 years, before inclusion in the COHADO cohort (in this group, the date of disclosure was often not recorded).

Second, we compared the baseline characteristics of APHIV according to their 24-month HIV disclosure status. Among those who were unaware of their HIV status at inclusion, we analyzed the factors associated with HIV disclosure during the follow-up period using a logistic regression. The person involved in the HIV disclosure process, timing of HIV disclosure regarding ART initiation, and reasons for non-disclosure were described.

Third, we described the 24-month clinical, immunological, and virological outcomes both individually and as a combined outcome. The combined 24-month outcome was measured by combining multiple criteria: (i) vital status [alive, died, lost to follow-up (defined last clinical contact >6 months, and for whom transfer, or vital status was unknown at database closure)]; (ii) WHO AIDS stage progression since baseline [WHO Clinical staging of HIV is a symptomatic classification of HIV that ranges according to increasing severity from 1 to 4 (36)]; (iii) immunological criteria (% of CD4 cell count difference since baseline); and (iv) virological criteria (HIV viral load measured). An unfavorable outcome was defined when at least one of the following events occurred: death, loss to follow-up, progression to AIDS clinical stage during the study period, a CD4 count decrease >10% compared to baseline, or a detectable viral load >50 copies/mL. If one of the evaluation criteria was missing to determine this combined outcome, adolescents were nevertheless classified using the remaining documented criteria. A favorable 24-month outcome was therefore defined as none of the above events occurring over the study period. Correlates of a 24-month favorable combined outcome, according to the HIV disclosure status and other baseline variables, were described using a full logistic regression model. The co-variable that was significantly associated with a 20% threshold in the univariate analysis were included in the adjusted logistic regression model accounting for country-specific practices regarding HIV-disclosure. We also performed a sub-analysis investigating the correlates of a 24-month favorable combined outcome in a sub-population of APHIV who had their viral load data available at 24 months. Model fits were checked graphically using Pearson residuals.

Categorical variables were described using counts and percentages and compared using the Pearson's Chi^2^ tests (or Fisher's Exact test if adapted). Continuous variables were described by median and interquartile ranges (IQR) and compared using the Wilcoxon-Mann-Whitney test. All analyses were performed using STATA 14.2 (Statacorp, College Station, TX, USA), with a 5% significance level.

## Results

### Selection and Inclusion of APHIV in the COHADO Cohort

**Figure 1:**
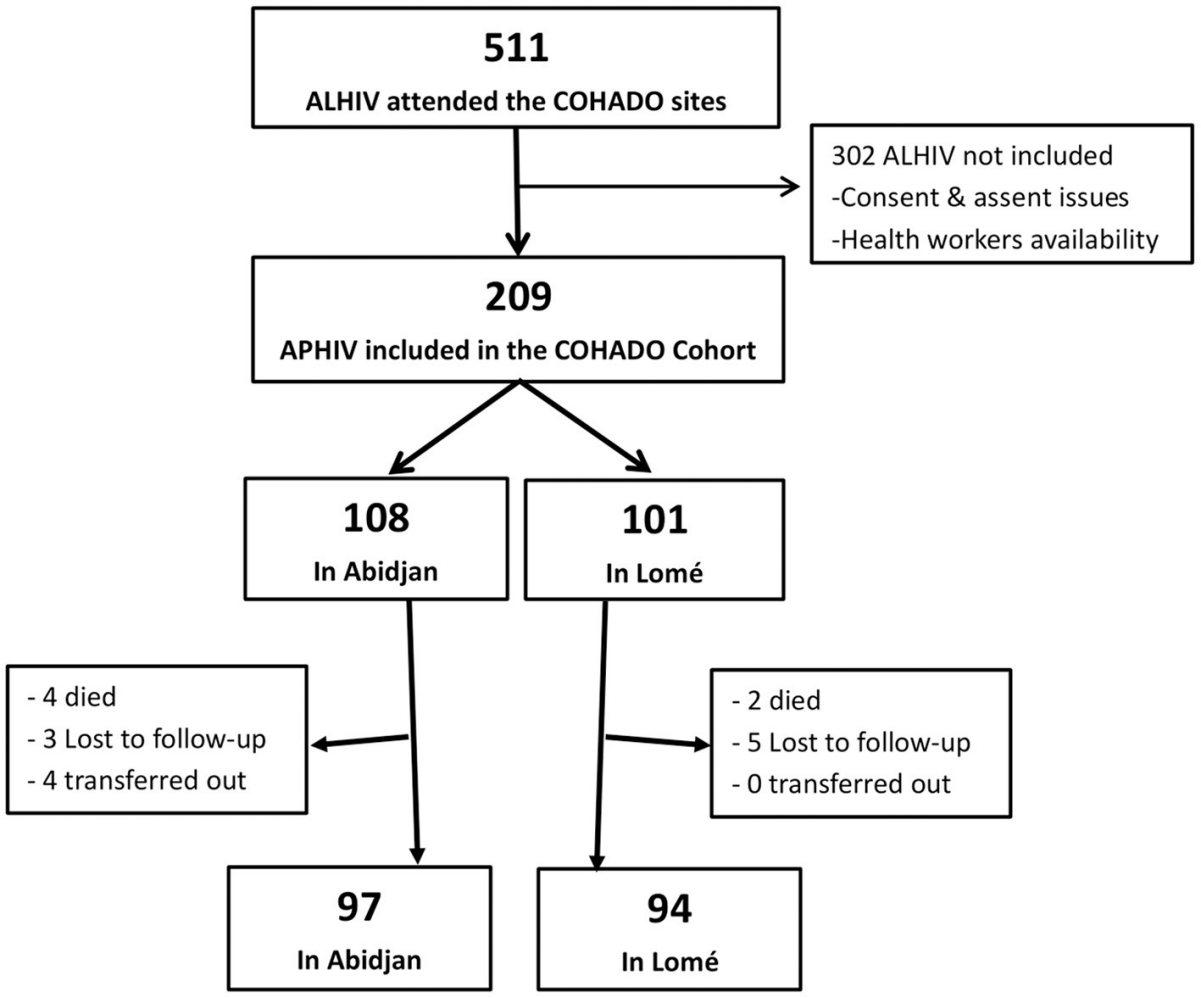
Flow chart of the 209 APHIV participant in the COHADO Cohort, IeDEA-pWADA, 2015.

From January to November 2015, 511 APHIV visited the sites of Abidjan and Lomé. Among them, a total of 209 (40.9%) APHIV were offered enrollment in COHADO and gave their consent (Figure 1). Reasons for not being included were mainly due to either parent and APHIV refusal or low availability of health care workers to enroll in the study, due to work overload. This was significantly more observed in Abidjan compared to Lomé (Table 1). ALHIV who were included in COHADO did not differ from those who were not included, in terms of sex, age, and immunological status distributions at baseline. However, at inclusion, there were significantly higher rates of missing data for the WHO AIDS clinical staging and viral load among those who were not included compared to those who were included in the COHADO cohort (Table 1).

**Table 1:** Characteristic of eligible adolescents living with perinatally-acquired HIV (APHIV) included in the COHADO cohort compared to those who were not included, during the inclusion period in Abidjan (Côte d'Ivoire), Lomé (Togo), IeDEA-pWADA, 2015–2017.

	**Total**	**Not included in COHADO**	**Included in COHADO**	***P*- Value**
	***N* = 511 (%)**	***N* = 302 (%)**	***N* = 209 (%)**	
**Site**, ***n*** **(%)**				<0.01^a^
Abidjan (Côte d'Ivoire)	371 (72.6)	263 (87.1)	108 (51.7)	
Lomé (Togo)	140 (27.4)	39 (12.9)	101 (48.3)	
**Sex**, ***n*****(%)**				0.61^a^
Males	239 (46.8)	144 (47.7)	95 (45.4)	
Females	272 (53.2)	158 (52.3)	114 (54.6)	
**Age in years, median [IQR]**	13 [11–15]	14 [12–16]	13 [11–15]	0.06^c^
**Age initiation ART**,				
**median [IQR]**	7[4–9]	6[4–9]	7[4–10]	0.12^c^
**WHO clinical stage**				<0.01^b^
1, 2, 3	380 (74.4)	207 (68.5)	173 (82.8)	
4 (AIDS)	43 (8.4)	7 (2.3)	36 (17.2)	
Missing	88 (17.2)	88 (29.4)	0 (0.0)	
**Virological suppression at last**, ***n*** **(%)**				
**(Viral load <50 cp/mL)**				<0.01^b^
Yes	34 (6.7)	32 (10.6)	2 (1.0)	
No	190 (37.2)	130 (43.0)	60 (28.7)	
Missing	287 (56.1)	140 (46.4)	147 (70.3)	
**CD4, median [IQR]**	483 [258–701]	487 [277–678]	534 [278–798]	0.76^c^

a *Chi^2^ test;*

b *Fisher's exact test;*

c *Wilcoxon-Mann-Whitney test*.

### Baseline Characteristics

Baseline characteristics are presented in Table 2. Among the 209 APHIV included, 108 (52%) lived in Abidjan. Overall, 114 (55%) were females and the median age was 13 years (IQR: 11–15). APHIV were significantly older in Abidjan (14 years) compared to Lomé (12 years, *p* = 0.01). Median ART duration prior to inclusion in the COHADO cohort was 6 years (IQR: 4–10), and it was significantly longer in Abidjan [9, IQR: (7–11)] compared to Lomé [5, IQR: (2–6)] (*p* <0.01). Only 23.9% of the cohort lived with both their biological parents. Most APHIV (89.9%) were from urban areas, 97.1% had access to electricity, and 62.7% had access to running water at home.

**Table 2:** Baseline characteristics of the 209 adolescents living with perinatally-acquired HIV included in the COHADO cohort according to sites, Abidjan (Côte d'Ivoire), Lomé (Togo) IeDEA-pWADA, 2015–2017.

	**Total**	**Abidjan, Côte d'Ivoire**	**Lomé, Togo**	***P*-Value^**a**^**
	***N* = 209 (%)**	***N* = 108 (%)**	***N* = 101 (%)**	
**Sex** ***n*** **(%)**				0.25
Males	95 (45.4)	45 (41.7)	50 (49.5)	
Females	114 (54.6)	63 (58.3)	51 (50.5)	
**HIV disclosure performed**, ***n*** **(%)**				<0.01
Yes	87 (41.6)	61 (57.4)	25 (24.8)	
No	122 (58.4)	45 (42.6)	76 (75.2)	
**Education level**, ***n*** **(%)**				0.01
Primary school	80 (38.3)	31 (28.7)	49 (48.5)	
Middle schools	103 (49.3)	61 (56.5)	42 (41.6)	
High schools	26 (12.4)	16 (14.8)	10 (9.9)	
**Residential environment**, ***n*** **(%)**				0.91
Urban	187 (89.9)	96 (88.9)	91 (90.1)	
Rural	22 (10.1)	12 (11.1)	10 (9.9)	
**Electricity access**, ***n*** **(%)**				0.43^b^
Yes	203 (97.1)	106 (98.1)	97 (96.0)	
No	6 (2.9)	2 (1.9)	4 (4.0)	
**Running water access**, ***n*** **(%)**				<0.01
Yes	131 (62.7)	101 (93.5)	30 (29.7)	
No	78 (37.3)	7 (6.5)	71 (70.3)	
**Living with**, ***n*** **(%)**				0.65
Both parents	50 (23.9)	27 (25.0)	23 (22.8)	
Only one parent	93 (44.5)	50 (46.3)	43 (42.6)	
Other family member	66 (31.6)	31 (28.7)	35 (34.6)	
**Orphanhood**, ***n*** **(%)**				0.03
No	91 (43.5)	44 (40.7)	47 (46.5)	
One parent	91 (43.5)	55 (50.9)	36 (35.6)	
Two parents	27 (13.0)	9 (8.4)	18 (17.8)	
**WHO clinical stage**, ***n*** **(%)**				<0.01
1, 2, 3	173 (83.8)	105 (97.2)	68 (67.3)	
4 (AIDS)	36 (17.2)	3 (2.8)	33 (32.7)	
**ART regimen**, ***n*** **(%)**				0.48
2 NRTI+1NNRTI	168 (81.2)	88 (83.0)	80 (79.2)	
2 NRTI+1PI	39 (18.8)	18 (17.0)	21 (20.8)	
**Good ART adherence**^**†**^, ***n*** **(%)**				<0.01^b^
Yes	118 (56.4)	57 (52.7)	61 (51.6)	
No	57 (27.3)	24 (22.2)	33 (32.6)	
Missing	34 (16.3)	27 (25.0)	7 (6.9)	
**Virological suppression**, ***n/N*** **(%)**				
**(viral load** ** <50 cp/mL)**				<0.01
Yes	2/62 (3.2)	0/53 (0.0)	2/9 (22.2)	
No	60/62 (96.8)	53/53 (100.0)	7/9 (77.8)	
**CD4 cell count/mm3, median [IQR]**				
	521[281–757]	540[314–753]	484[271.5–760]	0.71^c^
**Age at inclusion in years, median [IQR]**				
	13[11–15]	14[12–15]	12[11–15]	0.01^c^
**ART duration in years, median [IQR]**				
	6[4–10]	9[7–11]	5[2–6]	<0.01^c^
**Age at ART initiation, median [IQR]**				
	7[4–10]	5[3–8]	9[6–11]	<0.01^c^

a *Chi^2^ test;*

b *Fisher's exact test;*

c *Wilcoxon-Mann-Whitney test.*

† *More than 95% of planned doses taken. ART, Antiretroviral therapy*.

Overall, 17.2% had already reached the WHO AIDS stage 4 at baseline. This proportion was significantly higher in Lomé (32.7%) compared to Abidjan (2.8%) (*p* < 0.01). Regarding ART, 81.3% were receiving a NNRTI-based regimen, and 56.4% of APHIV showed good adherence to ART, as defined in the Methods section. Only 29.6% of APHIV had a viral load measurement; of whom, 3.2% with virological suppression (<50 cp/mL). At baseline, 41.6% were already fully HIV-disclosed. This proportion was significantly higher in Abidjan (57.4%) compared to Lomé (24.8%, *p* < 0.01).

### HIV Disclosure Characteristics and Process

Among the 209 APHIV enrolled, 122 (58.4%) were not informed of their HIV status at inclusion in the COHADO cohort, while they were aged of 12 years in median. Questioned about the reasons of non-disclosure at inclusion, parents/legal guardians declared fear of the adolescent's reaction (75.0%), and fear of HIV status disclosure to others (69.1%) were the most frequent reasons. Other reasons included the young age of APHIV (52.9%) and the fact that their parents were not prepared (35.3%).

At the 24-month endpoint, 87 (41.6%) APHIV were informed of their HIV status before inclusion, 68 (32.5%) were disclosed to during follow-up, and 54 (25.8%) were still not aware. Baseline characteristics of APHIV according to the HIV disclosure status at the endpoint are presented in Table 3. Overall, 74.1% were fully HIV-disclosed at the 24-month endpoint (+43.9% relative increase compared to baseline, *p* < 0.01).

**Table 3:** Baseline characteristics of the 209 APHIV included according to HIV-serostatus disclosure at 24-month, in the COHADO cohort, Abidjan (Côte d'Ivoire), Lomé (Togo), IeDEA-pWADA, 2015–2017.

	**Total** ***N* = 209 (%)**	**HIV-status disclosed before** **inclusion in COHADO** ***N* = 87 (%)**	**HIV-status disclosed during follow-up in COHADO** ***N* = 68 (%)**	**HIV-status still not disclosed at the last follow-up** ***N* = 54 (%)**	***P*-Value^**a**^**
**Sex**, ***n*** **(%)**					0.64
Males	95 (45.4)	37 (42.5)	34 (50.0)	24 (44.4)	
Females	114 (54.6)	50 (57.5)	34 (50.0)	30 (55.6)	
**Site**, ***n*** **(%)**					<0.01
Abidjan (Côte-d'Ivoire)	108 (51.7)	62 (71.3)	15 (22.1)	31 (57.4)	
Lomé (Togo)	101 (48.3)	25 (28.7)	53 (77.9)	23 (42.6)	
**Age at baseline in years, median [IQR]**	13[11–15]	15[14–17]	12[11–14]	11[10–12]	<0.01^c^
**Orphanhood**, ***n*** **(%)**					0.36
No	91 (43.5)	31 (35.6)	31 (47.1)	28 (51.6)	
One parent	91 (43.5)	43 (49.4)	27 (39.7)	21 (38.9)	
Two parents	27 (13.0)	13 (14.9)	9 (13.2)	5 (9.3)	
**Education level**					<0.01^b^
Primary school	80 (38.3)	14 (16.1)	31 (45.6)	35 (64.8)	
Middle schools	103 (49.3)	49 (56.3)	35 (51.5)	19 (35.2)	
High schools	26 (12.4)	24 (27.6)	2 (2.9)	0 (0.0)	
**Running water access**					<0.01
Yes	131 (62.7)	67 (77.0)	32 (47.1)	32 (59.3)	
No	78 (37.3)	20 (22.9)	36 (52.9)	22 (40.7)	
**Electricity access**, ***n*** **(%)**					0.76
No	6 (2.9)	3 (3.4)	1 (1.5)	2 (3.7)	
Yes	203 (97.1)	84 (96.6)	67 (98.5)	52 (96.3)	
**Residential environment**					0.53
Urban	187 (89.5)	77 (88.5)	63 (92.6)	47 (87.1)	
Rural	22 (10.5)	10 (11.5)	5 (7.4)	7 (12.9)	
**Living with**					0.94
Both parents	50 (23.9)	19 (21.8)	18 (26.5)	13 (24.1)	
Only one parent	93 (44.5)	38 (43.7)	30 (44.1)	25 (46.3)	
Other family member	66 (31.6)	30 (34.5)	20 (29.4)	16 (29.6)	
**WHO clinical stage**, ***n*** **(%)**					0.41^b^
1, 2, 3	173 (82.8)	73 (83.9)	53 (77.9)	47 (87.1)	
4 (AIDS)	36 (17.2)	14 (16.1)	15 (22.1)	7 (12.9)	
**Virological suppression (*****n*** **=** **62)**,					
**(Viral load** ** <50 cp/mL)**					0.68^b^
Yes	2 (3.2)	1 (2.9)	0 (0.0)	1 (6.3)	
No	60 (97.8)	34 (97.1)	11 (100.0)	15 (93.7)	
**CD4 cell count/mm3, median [IQR]**					
	521[281–758]	492[225–734]	507[262–850]	548[416–843]	0.08^c^

a *Chi^2^ test;*

b *Fisher's exact test;*

c *Kruskal-Wallis test*.

Among the 122 APHIV who were unaware of their HIV status at inclusion, those who were informed of their HIV status at the end of follow-up tended to have a lower CD4 cell count at baseline compared to those who were not HIV-disclosed to (Table 3). Compared with APHIV who were not HIV-disclosed, APHIV who were HIV-disclosed during the study were significantly older [13 years at baseline IQR (11–14) vs. 11 years IQR (10–12), *p* < 0.01], on ART for a shorter period of time [5 years IQR (2–8) vs.0 7 years IQR (5–9), *p* = 0.03], and mostly from Lomé (78 vs. 43%, *p* > 0.01). Adjusted for covariates (sex, education level, residential environment, person with whom APHIV lives, baseline clinical AIDS stage, CD4 cell count, age, and ART duration), APHIV from Lomé were significantly more likely to be HIV-disclosed during the study compared to those from Abidjan [adjusted odds ratio (aOR): 15.2, 95% CI (3.12–73.9)] (Table 4).

**Table 4:** Correlates of HIV disclosure at 24-month among the 122 APHIV not disclosed at inclusion, in the COHADO cohort, Abidjan (Côte d'Ivoire), Lomé (Togo), IeDEA-pWADA, 2015–2017.

	**Univariate analysis**				**Multivariate analysis**		
	**Total** ***N* = 122 (%)**	**HIV status disclosed** ***N* = 68 (%)**	**HIV status not disclosed** ***N* = 54 (%)**	***P*-Value^**a**^**	**Full model**		
					**aOR**	**(95% CI)**	***p-*value**
**Sex**, ***n*** **(%)**				0.54			
Males	58 (47.5)	34 (50.0)	24 (44.4)		1.00	—	—
Females	64 (52.5)	34 (50.0)	30 (55.6)		1.02	(0.41–2.51)	0.96
**Site**, ***n*** **(%)**				<0.01			
Abidjan (Côte-d'Ivoire)	46 (37.7)	15 (22.1)	31 (57.4)		1.00	—	—
Lomé (Togo)	76 (62.3)	53 (77.9)	23 (42.6)		15.2	(3.12–73.9)	<0.01
**Orphanhood**, ***n*** **(%)**				0.76			
No	60 (49.2)	32 (47.1)	28 (46.5)				
One parent	48 (39.3)	27 (39.7)	21 (35.6)				
Two parents	14 (11.5)	9 (13.2)	5 (17.8)				
**Education level**				0.05			
Primary school	66 (54.1)	31 (45.6)	35 (64.8)		1.00	—	—
Middle schools	54 (44.3)	35 (51.5)	19 (35.2)		1.42	(0.49–4.16)	0.51
High schools	2 (1.6)	2 (2.9)	0 (0.0)		1.00	—	—
**Running water access**				0.18			
Yes	64 (52.4)	32 (47.1)	32 (59.3)		1.00	—	—
No	58 (47.5)	36 (52.9)	22 (40.7)		0.51	(0.11–2.12)	0.35
**Electricity access**, ***n*** **(%)**				0.58			
Yes	119 (97.5)	67 (98.5)	52 (96.3)				
No	3 (2.46)	1 (1.5)	2 (3.7)				
**Residential environment**				0.36			
Urban	110 (90.2)	63 (92.6)	47 (87.1)		1.00	—	—
Rural	12 (9.8)	5 (7.4)	7 (12.9)		0.62	(0.11–3.27)	0.58
**Living with**				0.95			0.93
Both parents	31 (25.4)	18 (26.5)	13 (24.1)		1.00	—	—
Only one parent	55 (45.1)	30 (44.1)	25 (46.3)		1.17	(0.37–3.67)	0.78
Other family member	36 (29.5)	20 (29.4)	16 (29.6)		0.99	(0.27–3.61)	0.98
**WHO clinical stage**, ***n*** **(%)**				0.19^b^			
1, 2, 3	100 (81.9)	53 (77.9)	47 (87.1)		1.00	—	—
4 (AIDS)	22 (18.1)	15 (22.1)	7 (12.9)		0.44	(0.10–1.84)	0.26
**CD4 cell count/mm**^**3**^**, median [IQR]**						
	546[305–843]	508[262–850]	548[416–843]	0.19 ^c^	0.99	(0.99–1.00)	0.88
**Virological suppression (*****n*** **=** **27)**,						
**(Viral load** ** <50 cp/mL)**			1.00			
Yes	1 (3.7)	0 (0.0)	1 (6.3)				
No	26 (96.3)	11 (100.0)	15 (93.7)				
**Age at inclusion in years, median [IQR]**							
	12[11–13]	13[11–14]	11[10–12]	<0.01^c^	1.86	(1.31–2.63)	<0.01
**Age at ART initiation, median [IQR]**							
	6[4–9]	8[4–11]	5[3–7]	<0.01^c^			
**ART duration in years, median [IQR]**							
	6[3–9]	5[2–8]	7[5–9]	0.03^c^	0.96	(0.80–1.15)	0.69

a *Chi^2^ test;*

b *Fisher's exact test;*

c *Wilcoxon-Mann-Whitney test*.

Among the 155 APHIV who were fully HIV-disclosed to by 24 months, both parents (40.7%) and psychologists (54.2%) were the most involved in the HIV disclosure process, doctors were involved in the process for only 5.2% of APHIV, and counselors for 1.9% (Table 5).

**Table 5:** Characteristics of the HIV disclosure modalities to the 155 adolescents living with perinatally-acquired HIV disclosed at 24 months by sites in the COHADO cohort, IeDEA-pWADA, 2015–2017.

	**Total**	**Abidjan, Côte d'Ivoire**	**Lomé, Togo**
	***N* = 155 (%)**	***N* = 77 (%)**	***N* = 78 (%)**
**Person involved in HIV disclosure**, ***n*** **(%)**			
Mother	41 (26.5)	30 (43.5)	11 (14.3)
Father	22 (14.2)	18 (26.1)	4 (5.2)
Doctor	8 (5.2)	6 (8.7)	2 (2.6)
Psychologist	84 (54.2)	25 (36.2)	59 (76.6)
Counselor	3 (1.9)	0 (0.0)	3 (2.1)
Other (Family or association members)	3 (1.9)	0 (0.0)	3 (2.1)
**Disclosure performed before ART initiation**			
Yes	13 (8.4)	6 (7.8)	7 (8.4)
No	133 (85.8)	64 (83.1)	69 (88.5)
Unknown	9 (5.8)	7 (9.1)	2 (2.6)

### 24-Month Health Outcomes

By 24 months, six APHIV had died (2.9%) and eight were lost to follow-up (3.8%). Among the remaining APHIV followed-up, 71.7% (150/197) were still on a NNRTI-based regimen.

By 24 months, 99.0% (207/209) of the APHIV had their clinical data available, of whom, 54.5% were classified as having an “unfavorable” outcome; 99.0% (207/209) had their immunological data available, of whom, 55.0% were classified as “unfavorable”; and 83.7% (175/209) had their virological data available, of whom, 61.1% were classified as “unfavorable.” Altogether, 175 (83.7%) had their data available for all components used to build the combined outcome at 24 months and 34 (16.3%) had at least their viral load data missing.

The proportion of APHIV who were HIV-disclosed during the follow-up period was higher in Lomé (52.5%) than in Abidjan (13.9%) (*p* < 0.01) (Table 6). Among the 209 APHIV, the 24-month combined health outcome was favorable for 45% of APHIV overall. This proportion was significantly higher in Lomé (61.4%) compared to Abidjan (29.6%, *p* < 0.01) (Table 6).

**Table 6:** 24-month component and combined outcomes of the 209 adolescents living with perinatally-acquired HIV included, by sites in the COHADO cohort, IeDEA-pWADA, 2015–2017.

	**Total**	**Abidjan, Côte d'Ivoire**	**Lomé, Togo**	***P*-Value^**a**^**
	***N* = 209 (%)**	***N* = 108 (%)**	***N* = 101 (%)**	
**HIV-disclosure performed**, ***n*** **(%)**				** <0.01**
No	54 (25.9)	31 (28.7)	23 (22.7)	
Yes (Since ≤ 2 years)	68 (32.5)	15 (13.9)	53 (52.5)	
Yes (Since >2 years)	87 (41.6)	62 (57.4)	25 (24.8)	
**Follow-up**, ***n*** **(%)**				0.18^b^
Died	6 (2.9)	4 (3.7)	2 (2.0)	
Lost to follow-up	8 (3.8)	3 (2.8)	5 (4.9)	
Transferred out	4 (1.9)	4 (3.7)	0 (0.0)	
Alive & follow-up	191 (91.4)	97 (89.8)	94 (93.1)	
**Progression to AIDS WHO**				
**Stage during the study period**, ***n*** **(%)**				** <0.01**^**b**^
Yes	7 (3.3)	5 (4.6)	2 (2.0)	
No	154 (73.7)	91 (84.2)	63 (62.4)	
Already AIDS WHO at inclusion	36 (17.3)	3 (2.8)	33 (32.7)	
Died, LTFU, transferred out, missing	12 (5.7)	9 (8.3)	3 (2.9)	
**CD4 count decrease** **≥** **baseline**				
**value (±10%)**, ***n*** **(%)**				** <0.01**
Yes	76 (36.3)	55 (51.4)	21 (21.0)	
No	131 (62.7)	52 (48.6)	79 (69.0)	
Missing	2 (1.0)	1 (1.0)	1 (1.0)	
**Viral load detectable at 24-month**				
**(>50 cp/mL)**				**0.01**
Yes	73 (34.9)	56 (51.8)	17 (16.8)	
No	102 (48.8)	49 (45.4)	53 (52.5)	
Missing	34 (16.3)	3 (2.8)	31 (30.7)	
**Combined outcome**^**†**^, ***n*** **(%)**				** <0.01**
Unfavorable	115 (55.0)	76 (70.4)	39 (38.6)	
Favorable	94 (45.0)	32 (29.6)	62 (61.4)	

a *Chi^2^ test;*

b *Fisher's exact test.*

† *Unfavorable combined outcome at 24 months: death or progression to AIDS during the study period or CD4 count decrease >10% compared to baseline or detectable viral load.*

Favorable outcome: none of the outcomes defined above notified.

*The bold values are statistically significants with a p value < 5%*.

Table 7 presents the correlates of a 24-month favorable combined health outcome in APHIV. In the univariate analysis, we found that APHIV from Lomé [vs. Abidjan, OR = 3.77, 95% CI (2.12–6.71), *p* < 0.01] and those without access to running water [vs. with access to running water, OR = 2.09, 95% CI (1.18–3.70), *p* = 0.01] were significantly more likely to have a favorable outcome. Age at inclusion was not associated with the outcome. Longer treatment duration reduced the probability of having a 24-month favorable outcome [OR = 0.90 per year, 95% CI (0.83–0.97), *p* = 0.01].

**Table 7:** Correlates of a favorable combined 24-month health outcome (definition: Table 5) of the 209 APHIV included, in the COHADO, IeDEA-pWADA, 2015–2017.

	**Univariate analysis**					**Adjusted analysis**		
	**Unfavorable combined outcome**	**Favorable combined outcome**						
	***n* = 115 (%)**	***n* = 94 (%)**	**OR**	**(95% CI)**	***p*-value**	**aOR**	**(95% CI)**	***p*-value**
**HIV-Disclosure**					0.17			
No	27 (23.5)	27 (28.7)	1.00	—	—	—	—	—
Yes (Since ≤ 2 years)	35 (30.4)	33 (35.1)	0.94	(0.46–1.92)	0.87	—	—	—
Yes (Since >2 years)	53 (46.1)	34 (36.2)	0.64	(0.32–1.27)	0.20	—	—	—
**Site**								
Abidjan, Côte-d'Ivoire	76 (66.1)	32 (34.1)	1.00	—	—	—	—	—
Lomé, Togo	39 (33.9)	62 (65.9)	3.77	(2.12–6.71)	** <0.01**	—	—	—
HIV Site*Disclosure (exploratory Interaction)					** <0.01**			** <0.01**
Abidjan, Côte-d'Ivoire								
HIV not disclosed	23 (11.0)	8 (3.8)	1.00	—	—	1.00	—	—
HIV disclosed ≤ 2years	13 (6.2)	2 (0.9)	0.44	(0.08–2.40)	0.34	0.34	(0.06–1.92)	0.22
HIV disclosed >2years	40 (19.1)	22 (10.5)	1.58	(0.61–4.12)	0.35	1.44	(0.53–3.87)	0.47
Lomé, Togo								
HIV not disclosed	4 (1.9)	19 (9.1)	1.00	—	—	1.00	—	—
HIV disclosed ≤ 2 years	22 (10.5)	31 (14.8)	0.29	(0.08–0.99)	**0.04**	0.30	(0.87–1.04)	0.05
HIV disclosed >2 years	13 (6.2)	12 (5.7)	0.19	(0.05–0.73)	**0.02**	0.21	(0.05–0.84)	**0.03**
**Sex**								
Males	48 (41.7)	47 (50.0)	1.00	—	—	1.00	—	—
Females	67 (58.3)	47 (50.0)	0.71	(0.41–1.23)	0.23	0.76	(0.41–1.41)	0.39
**Baseline ART regimen**, ***n*** **(%)**								
2 NRTI+1PI	19 (33.9)	20 (21.3)	1.00	—	—			
2 NRTI+1NNRTI	94 (66.1)	74 (78.7)	0.75	(0.37–1.50)	0.42			
**Education level**					0.63			
Primary school	41 (35.6)	39 (41.5)	1.00	—	—			
Middle schools	60 (52.2)	43 (45.7)	0.75	(0.41–1.35)	0.34			
High schools	14 (12.2)	12 (12.8)	0.90	(0.37–2.18)	0.81			
**Running water access**								
Yes	81 (70.4)	50 (53.2)	1.00	—	—	1.00	—	—
No	34 (29.6)	44 (46.8)	2.09	(1.18–3.70)	0.01	0.60	(0.25–1.45)	0.26
**Electricity access**, ***n*** **(%)**								
Yes	112 (97.4)	91 (96.8)	1.00	—	—			
No	3 (2.6)	3 (3.2)	1.23	(0.24–6.24)	0.80			
**Residential environment**								
Urban	100 (86.9)	87 (92.6)	1.00	—	—	1.00	—	–
Rural	15 (13.1)	7 (7.4)	0.53	(0.21–1.37)	0.19	0.64	(0.23–1.81)	0.40
**Living with**					0.35			
Both parents	25 (21.7)	25 (26.6)	1.00	—	—	1.00	—	—
Only one parent	49 (42.6)	44 (46.8)	0.89	(0.45–1.78)	0.75	0.99	(0.45–2.15)	0.98
Other family member	41 (35.7)	25 (26.6)	0.60	(0.28–1.28)	0.19	0.56	(0.24–1.29)	0.17
**Age at baseline, median [IQR]**	13 [11–15]	13[11–15]	0.96	(0.86–1.07)	0.54			
**ART duration in years, median [IQR]**	8[4–10]	7[4–10]	0.90	(0.83–0.97)	0.01	0.98	(0.89–1.09)	0.52

*OR: Odds Ratio aOR:adjusted Odds Ratio. The bold values are statistically significants with a p value < 5%*.

There was a significant interaction between HIV disclosure and country. In the multivariate analyses adjusted for sex, access to running water, residential environment, and being orphan or not, HIV disclosure for >2 years significantly reduced the probability of having a favorable 24-month outcome compared to those who were not HIV-disclosed in Lomé [Disclosed > 2 years: aOR = 0.21, 95% CI (0.05–0.84), *p* = 0.03], while there was no significant association in Abidjan (Table 7). Access to running water and ART duration were the confounding factors that were no longer significant in the adjusted analysis. A sensitivity analysis investigating the correlates of a 24-month favorable combined outcome restricted to adolescents with available data for all criteria was run, but this did not change the main results (data not shown).

## Discussion

To our knowledge, this is the first cohort to report a snapshot of the clinical, immunological, and virological outcomes of APHIV measured over 24 months in relation to HIV disclosure in two West African pilot sites contributing to the IeDEA West Africa collaboration. We made several key findings highlighting the field reality of the evolving trends of APHIV care from 2015 to 2017. First, full HIV disclosure status to APHIV increased from 46 to 74% after 24 months of follow-up, but we still found that one in four APHIV remained not formally disclosed to by the time of the endpoint, while they were all aged above 13 years. Second, unlike caregivers and psychologists, doctors and counselors have a very little involvement in the disclosure process. Third, although access to viral load increased from 30% at inclusion to 84% after 24 months, still, 16% had no viral load measurement. Fourth, we found that after 24 months of follow-up, the cumulative death rate was high, close to 3%, and only 45% of APHIV had a favorable 24-month combined outcome, while 77.5% were still receiving a NNRTI-based regimen. Finally, in the adjusted analysis of the correlates of a combined health outcome, we found that being HIV-disclosed before the inclusion was a significant marker of a worse outcome in Lomé, while there was no association found in Abidjan.

Although the frequency of ALHIV fully HIV-disclosed differs among African studies, it remains low overall, ranging from 16 to 39% (37–42). In our study, we found that only 46.1% knew their HIV status at baseline and 74.2% by 24 months of follow-up. Although this should be closer to 100% according to the WHO recommendations, our result is higher than that reported in previous studies, particularly in West Africa (26, 32, 43). In Ghana, two separate studies reported the proportion of HIV disclosure to be 11.2% among children and adolescents aged 8–14 years in 2009 and 44% among ALHIV aged 12–19 years in 2015 (26, 44). Furthermore, we observed an increase in the HIV disclosure process over the 24-month follow-up period. This is clearly visible in Lomé where the proportion of APHIV disclosed increased from 24.8 to 77.2%. APHIV from Lomé were more likely to be disclosed during the study, due to the low disclosure rates at baseline, younger age, and older age at ART initiation in this cohort. This increase could indeed mainly be explained by the cohort effect, as adolescents age over time, and age is one of the main factors of HIV disclosure. However, in the COHADO context, this is also likely the consequence of a 3-day training workshop on the HIV-serostatus disclosure to ALHIV delivered in 2016, involving HIV health professionals from all the IeDEA-pWADA sites (45). West and Central African healthcare workers (doctors, psychologists, and social workers) and four expert adolescents living with HIV were invited to participate in a 3-day workshop to promote and support the HIV disclosure process in pediatric clinical sites. After sharing their own experiences about the HIV disclosure process, participants identified barriers and facilitating practices, including the participating adolescents' suggestions. A standardized disclosure process was then proposed to be implemented in their different facilities. This workshop involved the COHADO staff and may have changed the health care workers' perceptions, prompting their practices regarding HIV disclosure. As we were not able to document the rate of HIV disclosure before and after this workshop, we also acknowledge that we cannot isolate the effect of age from the training workshop effect to explain the HIV disclosure rate.

We found that few doctors are involved in the process of HIV disclosure and tended to delegate the HIV disclosure practice to other health care workers. Reasons for this included clinical work overload or fear in doing it. Indeed, health care workers face structural issues including limited human and technical resources, whereas disclosure is a complex process which needs time and training (22, 29). In 2018, there were too few counselors working in our HIV-programs. Thus, when available in HIV programs, psychologists are better trained to address mental issues such as HIV-related stigma and taboo, and therefore, are more involved in the HIV disclosure process. Consequently, they have become the only health care workers in charge of the full HIV disclosure process, but they are not always available on a daily basis. In our study, HIV disclosure was fortunately covered by psychologists, showing a privileged context. They were trained to HIV disclosure and developed a space for a better communication and a privileged relationship of trust with adolescents, constituting a central component for the issue of HIV disclosure. However, psychologists are scarce in resource-limited pediatric HIV care settings. In a study exploring the models of HIV disclosure in 180 HIV pediatric care sites, HIV disclosure counseling was most often provided by counselors (87% of sites), nurses (77%), physicians (74%), social workers (68%), or other clinicians (65%) (46). More recently, peer-educators have also been successfully involved. Therefore, we feel that the whole staff should be trained and involved in the HIV disclosure process with a multidisciplinary approach and task-sharing. That approach should be set up at each HIV program level to offer comprehensive care, including the process of HIV status disclosure. It is important for national and regional programs to locally tailor appropriate strategies to improve disclosure practice, such as the training of a multidisciplinary team on disclosure, as mentioned earlier (45). Furthermore, caregivers are also unprepared for HIV disclosure and fear of stigmatization. For many parents, their children are too young or are not ready to receive HIV disclosure (27, 30, 47). The role and benefits of having caregivers involved in the HIV disclosure process remain unclear. While some studies have suggested that caregivers are in a much better position to disclose the ALHIV status, others have reported that according to ALHIV, health care workers are better placed (48–50). Nevertheless, a better understanding of what refrains caregivers to disclose to their child is important to support them accordingly.

In our study, the 24-month cumulative death rate reached 3%.This is high but similar to that reported in other studies conducted in ALHIV in sub-Saharan Africa, ranging 4–6% (34, 51). We also reported a sub-optimal virological response in our population that is in line with the results reported in a previous study conducted in Lomé in 2016, with high rates of virological failure and drug resistance among APHIV (17). APHIV in Lomé were in a significantly poorer clinical and immunological condition at baseline compared to those from Abidjan, and therefore, were more likely to reach a favorable 24-month outcome. However, APHIV from Abidjan were significantly more in virological failure than those from Lomé at baseline. However, APHIV from Abidjan were significantly older (2 years in median), and treated for a longer period, highlighting a cohort effect in site differences. Although we were not able to document the different HIV sub-types, we do not hypothesize that this could explain the difference between the two sites. Our finding highlights rather historical differences in the care practices and resources available between West African cities, which need to be considered to adapt APHIV care delivery. Overall, only 45% of APHIV had a favorable combined 24-month outcome which is poor in terms of the quality of ART response. These outcomes are observed in a context where close to 80% of APHIV were receiving a NNRTI-based therapy at baseline, then, still 77.5% after 24 months, reflecting a limited access to a second line therapy common in West-Africa. In our study, the baseline ART regimen was not associated with the 24-month outcome. This is likely an indication bias in a context where those receiving a PI-based regimen as a second line therapy where those failing in ART.

Many studies have reported on how HIV disclosure improves ALHIV clinical, immunological, and virological outcomes as well as retention in care (29, 33, 34, 51, 52). The relationship between the time of HIV disclosure and the 24-month outcome was difficult to assess in our study because a substantial proportion of APHIV had already been disclosed to before the inclusion in the COHADO cohort with no documented HIV-disclosure date. There was a statistically significant interaction between the site and HIV disclosure timing, and we present our results accounting for this cohort effect. Indeed, ALHIV in Abidjan were older than those from Lomé, HIV-disclosure practices were more active during the follow-up in Lomé than in Abidjan, and ALHIV from Lomé were at a more advanced stage of their HIV disease at inclusion compared to those from Abidjan. We found that HIV disclosure before inclusion in the study in Lomé significantly reduced the odds of having a favorable 24-month outcome while there was no association found in Abidjan. We interpret this association by HIV-disclosure being the marker of an advanced HIV disease that may have prompted the disclosure process. This is in line with a previous reporting that ALHIV who were not disclosed of their HIV-serostatus increased virological failure by 5-fold (53). Unfortunately, we were unable to document the adverse incidents after HIV disclosure. Transient negative psychological effects and prolonged negative reactions that have required the health care workers and parents' support have been described in the literature (54, 55).

Our study met several limitations. First, we enrolled less than half of the APHIV who visited the sites during the inclusion period due to logistical issues, which were mainly related to health care workers overwork, with little time to enroll APHIV in the study and get formal parental consent. Although we reported no differences in the age, sex, and baseline immune status between those included and excluded, there was a significant difference in the participation rates between both sites (Lomé and Abidjan), with APHIV from Lomé more likely to be enrolled. In addition, the small sample size and relatively short follow-up period have limited the statistical power of our analysis. This flaw could have overshadowed an association between the 24-month health outcomes in APHIV and several variables such as age, sex, and other relevant variables. Second, the APHIV selected in our study were from urban teaching hospitals specialized in adolescent HIV care where APHIV are likely to receive a better standard of care compared to rural centers. This affects the representativeness of our results to all ALHIV in these countries but provides useful insights on the HIV care practices at a country level. Third, the study design and few time points during the follow-up period did not allow us to perform a longitudinal analysis. Fourth, our data are relatively old. Although the WHO guidelines have evolved recommending new drugs, including dolutegravir since 2019 as the preferred HIV treatment option in all populations, these drugs are not yet available in Côte d'Ivoire and Togo in 2020. Thus, we feel that despite the age of the data, our results reflect the current situation in West Africa, and advocate for more potent drugs. In addition, the COHADO cohort reported a small proportion of adolescents lost to follow-up, reflecting a high quality of follow-up in the selected sites. Our study provides the original data documenting the feasibility and, indirectly, the positive effect of accompanying actively the HIV disclosure process using health care worker training on this topic and routine monitoring of this crucial event.

Our results underscore important findings toward improving the care of APHIV in West Africa. Since 2016, WHO recommends HIV RNA viral load as the best indicator to evaluate the ART response and viral load is universally recommended, particularly for ALHIV who have a high risk of virological failure (53, 56). However, this routine access to viral load remains limited in the field level in West Africa. In a recent situation analysis study, viral load monitoring was available in 43% of the facilities across sub-Saharan Africa and only in 8% of the facilities in West Africa (57). COHADO is one of the first West African cohort studies to provide time trends in access to viral load in West African adolescents. In the COHADO clinical sites, annual viral load measurement is a recent opportunity that should be scaled up, as virological success could also be an indicator used to encourage treatment adherence and health outcomes of APHIV. Conversely, identifying the virological failure early enough would be helpful in reinforcing the treatment adherence more closely to re-suppress viral load in this vulnerable population. Despite a substantial increase in the access to viral load data from < 30% at inclusion to 84% at 24 months, we highlighted that still 16% have their routine viral load data missing.

Furthermore, access to new ARV drugs, often left behind in West Africa, remains crucial. As integrase inhibitors are already being rolled out in other regions of the world since 2017, West Africa still struggles to access protease inhibitors, and children and adolescents remain on NNRTI-based regimens despite the sub-optimal PMTCT exposure and consequential expected drug resistance emergence. In the COHADO cohort, 77.5% of APHIV were still receiving NNRTI-based regimens, while 55% were failing on these regimens. In addition, although we are not able to precisely document the frequency of HIV-2 co-infection in our study, we also remind that West Africa is endemic for HIV-2, described as having a natural resistance to NNRTIs (58, 59). Our results advocate for a prompt roll-out of more potent antiretroviral drugs accessible to West-African APHIV. This also implies improving access to HIV genotyping to better identify the most appropriate regimens in adolescents that have been exposed to several antiretroviral drugs regimens, despite the limited laboratory capacity and high test costs (60, 61).

## Conclusion

Our study assessed the HIV-disclosure process and the 24-month outcomes of APHIV in two West African urban clinical sites. Although the frequency of full HIV disclosure remains insufficient, this project has encouraged full HIV disclosure practices, and the effect of HIV disclosure on the 24-month outcome differed across sites, reflecting different standards of care. This study contributes to a better understanding of the disclosure process among APHIV. The disclosure of the APHIV status is a crucial step in APHIV care and the disclosure process should be initiated early, and not only for those at an advanced stage of the disease. HIV disclosure should be addressed as a dynamic process involving health care workers, counselors, and parents or legal guardian as key players. Urgent interventions are needed to support timely APHIV disclosure practices in both caregivers and health care workers. These interventions should be monitored over time and include caregivers and healthcare providers and counselors.

Overall, the findings of the COHADO study also highlight the insufficient response to the current treatment strategies. The 24-month outcome of APHIV was favorable for less than half of them. It is crucial to monitor the virological outcomes of ALHIV on lifelong ART closely. There is an urgent need to improve the treatment adherence, access to viral load monitoring, and access to a more potent ART in West African cities. Our results should guide HIV programs to optimize ALHIV quality of care.

Finally, our study provides a reference to a tailored context-specific intervention in West Africa. Cohort studies among ALHIV such as in pWADA offer unique opportunities to optimize monitoring, standardize data collection, and offer interventional packages of care to integrate all specific ALHIV issues including HIV disclosure, ART adherence, virological outcomes, drug resistance, but also mental and sexual health issues with the perspective of improving the 90-90-90 cascade of care targets in the ALHIV population.

## Data Availability Statement

The raw data supporting the conclusions of this article will be made available by the authors, without undue reservation.

## Ethics Statement

The studies involving human participants were reviewed and approved by -Comité de Bioéthique pour la recherche en Santé (Togo) -Comié National d'éthique des sciences de la vie et de la santé (Côte d'Ivoire). Written informed consent to participate in this study was provided by the participants' legal guardian/next of kin.

## The Iedea West African Collaboration

Site investigators and cohorts: Adult cohorts: Marcel Djimon Zannou, CNHU, Cotonou, Benin; Armel Poda, CHU Souro Sanou, Bobo Dioulasso, Burkina Faso; Fred Stephen Sarfo & Komfo Anokeye Teaching Hospital, Kumasi, Ghana; Eugene Messou, ACONDA CePReF, Abidjan, Côte d'Ivoire; Henri Chenal, CIRBA, Abidjan, Côte d'Ivoire; Kla Albert Minga, CNTS, Abidjan, Côte d'Ivoire; Emmanuel Bissagnene & Aristophane Tanon, CHU Treichville, Côte d'Ivoire; Moussa Seydi, CHU de Fann, Dakar, Senegal; Akessiwe Akouda Patassi, CHU Sylvanus Olympio, Lomé, Togo. Pediatric cohorts: Sikiratou Adouni Koumakpai-Adeothy, CNHU, Cotonou, Benin; Lorna Awo Renner, Korle Bu Hospital, Accra, Ghana; Sylvie Marie N'Gbeche, ACONDA CePReF, Abidjan, Ivory Coast; Clarisse Amani Bosse, ACONDA_MTCT+, Abidjan, Ivory Coast; Kouadio Kouakou, CIRBA, Abidjan, Côte d'Ivoire; Madeleine Amorissani Folquet, CHU de Cocody, Abidjan, Côte d'Ivoire; François Tanoh Eboua, CHU de Yopougon, Abidjan, Côte d'Ivoire; Fatoumata Dicko Traore, Hopital Gabriel Toure, Bamako, Mali; Elom Takassi, CHU Sylvanus Olympio, Lomé,Togo. Coordinating & data centers: ADERA, ISPED & INSERM U1219, Bordeaux, France: François Dabis, Renaud Becquet, Charlotte Bernard, Shino Chassagne Arikawa, Antoine Jaquet, Karen Malateste, Elodie Rabourdin, Thierry Tiendrebeogo. INSERM U1027, Toulouse, France: Désiré Dahourou, Sophie Desmonde, Julie Jesson, Valeriane Leroy. PACCI, CHU Treichville, Abidjan, Côte d'Ivoire: Didier Koumavi Ekouevi, Jean-Claude Azani, Patrick Coffie, Abdoulaye Cissé, Guy Gnepa, Apollinaire Horo, Christian Kouadio, Boris Tchounga.

## Author Contributions

MD conducted the analyses and wrote the first draft of paper under VL's supervision. SD, JJ, DD, KM, HA, EA, J-PR, and VL reviewed and edited the paper. KM and VL were involved in the database management, study design, and statistical analyses. VL was involved in the pediatric IeDEA cohort coordination and fund raising. UT, FT, UA-B, TB, and HA were in charge of the cohort of adolescents and the database recording in each country involved in the study. All authors have read and approved the final manuscript.

## Conflict of Interest

The authors declare that the research was conducted in the absence of any commercial or financial relationships that could be construed as a potential conflict of interest.
